# Tribocorrosion Behavior of Mg Alloys on Sliding Friction in Hank’s Balanced Salt Solution

**DOI:** 10.3390/ma19081513

**Published:** 2026-04-09

**Authors:** Eri Miura, Chihiro Shiraishi, Sachiko Hiromoto

**Affiliations:** 1 Department of Materials and Synchrotron Radiation Engineering, Graduate School of Engineering, University of Hyogo, 2167 Shosha, Himeji 671-2280, Hyogo, Japan; 2 Research Center for Structural Materials, National Institute for Materials Science (NIMS), 1-2-1 Sengen, Tsukuba 305-0047, Ibaraki, Japan; hiromoto.sachiko@nims.go.jp

**Keywords:** magnesium alloys, sliding wear, tribocorrosion, biomaterial, crevice corrosion

## Abstract

The tribocorrosion behavior of AZ31 and WE43 was investigated during sliding wear tests in Hank’s balanced salt solution (HBSS) and pure water. While wear volume increased monotonically with load in air and water, HBSS exhibited a distinct non-monotonic trend; the maximum material loss occurred at the minimum load (0.98 N) and decreased at 2.94 N before rising again. This indicates that at low loads, degradation is primarily driven by accelerated chemical dissolution (tribocorrosion) rather than by purely mechanical abrasion. The magnitude of wear followed the order [HBSS] > [air] > [water] in the low-load range (0.98–1.96 N), whereas it shifted to [air] > [HBSS] > [water] in the high-load range (2.94–5.88 N). A comparison of the wear rate of the alloys shows that the wear rate in HBSS differs from that in water, depending on the hardness of the substrate, similar to conditions in air. Notably, the specific wear rate decreased as test duration increased under low loads, further suggesting that corrosion-induced volume loss significantly outweighs mechanical wear in this regime. The static corrosion test revealed that volume loss during tribocorrosion was higher than that under static corrosion conditions. While the deposition of corrosion products affected net volume loss, chemical dissolution remained the primary driver of the observed wear trends at low loads. Electrochemical data from anodic polarization curves confirmed that the specimen tested under a 0.98 N load exhibited lower corrosion resistance. Mechanistically, it was suggested that Cl^−^ ions contributed to the overall increase in wear, while NaHCO_3_ specifically contributed to the increase in wear in the low-load range.

## 1. Introduction

Metallic materials are used as biomaterials in artificial joints, fracture fixation materials, bolts, and stents because they have superior mechanical properties, such as strength and toughness, compared to ceramic and polymer materials [1], which are implanted in the body to reconstruct the injured area and then are removed after healing. However, there are cases in which these implants are left in the body to avoid reoperation, and their long-term placement may cause inflammation or an allergic reaction. In addition, stents implanted in blood vessels cannot be removed, leading to restenosis and hindering stent reimplantation. It is desirable to eliminate these problems by using materials that degrade and disappear with healing.

Biodegradable materials are mainly made from ceramics and polymers with the same structure as those of biological tissues. Examples include polymers such as poly-*l*-lactic acid ([–C(CH_3_)HC(=O)O–]_n_), polyethylene glycol (HO-(CH_2_-CH_2_-O)_n_-H), and ceramics such as hydroxyapatite (HAp) and its precursor, b-tricalcium phosphate (b-TCP, Ca_3_(PO_4_)_2_), which is used for bone plates, stents, and artificial bone for relatively small load-bearing parts [2,3,4,5]. However, these polymeric and ceramic materials have the problems of insufficient strength and long degradation time, and there is a need for metallic materials with high strength that can be safely degraded in vivo and absorbed and eliminated in the body [6,7,8,9,10,11]. Iron-based, zinc-based, and magnesium-based alloys have been proposed as load-bearing biodegradable materials [3,4,5,12,13,14].

Both Mg and Fe are essential elements for living bodies, and Mg is more susceptible to corrosion with a low standard electrode potential of −2.35 V (vs. SHE) than Fe with that of −0.44 V. In addition, Mg has a high specific stress because of its low density (1.74 Mg/m^3^), low Young’s modulus (40–45 GPa), and cortical bone [15,16,17,18,19,20], which prevents stress shielding, a problem when metals are used in hard tissue prosthetic materials. To obtain appropriate mechanical properties and lower dissolution rates, industrial Mg alloys such as AZ31 (Mg-3Al-1Zn) and WE43 (Mg-4Y-3RE-1Zr) are being considered for applications as biomaterials [21]. The applications of Mg alloys as biomaterials are expected not only in orthopedics, such as fracture fixation screws, bone plates, and intramedullary nails, but also bioabsorbable stents in cardiology, staples in surgery, cancer treatment in oncology, and scaffolds in neurology, oncology, and tissue engineering [22,23]. As such, many studies are underway to apply Mg alloys as bioabsorbable materials for medical use [1,2,9,24,25,26,27], and it has been found that corrosion control is insufficient even for alloying [8,9,28,29,30]. HAp, TCP and other coatings on Mg alloys have been reported to be effective in controlling the dissolution rate during the healing period [31,32,33,34].

When metallic materials are used as bone plates and screws, with or without coating, friction and wear occur between the plate and screw at the joint and between the plate and bone [35,36]. In the worn area where the metal is exposed, corrosion and wear coincide, and the mass loss is accelerated [37]. The wear of materials in corrosive environments is called tribocorrosion. The amount of mass loss due to tribocorrosion is larger than the sum of the mass loss due to corrosion alone and due to wear alone [38,39], and the mass loss rate is assumed to be more significant. In other words, knowledge of the tribocorrosion behavior of Mg alloys in a corrosive environment in vivo is an essential factor when considering the adaptation of Mg as a biodegradable material, and it is crucial to understand the interaction between wear and corrosion and their mechanisms.

Therefore, in this study, sliding wear tests were performed in air, deionized water, and Hank’s balanced salt solution (HBSS) on AZ31 and WE43, which are used as biomaterials. The dead-load dependence of the friction coefficient, wear volume, and specific wear rate was investigated. The effects of corrosion and loading on the wear behavior of the Mg alloys were studied by surface observation and chemical composition analysis. The qualitative effects of different solutions, atmospheres, or loads on the wear parameters and wear surface morphologies were clarified. The tribocorrosion initiation mechanism and the factors that promote this phenomenon are discussed.

## 2. Experimental Procedures

### 2.1. Material Preparation

AZ31 and WE43 extrusions were used as the samples. The compositions of WE43 and AZ31 are presented in Table 1 and Table 2, respectively. The specimens were cut from *φ*15 mm or *φ*16 mm extruded bars to a thickness of 1.5 mm using a fine cutter and used as plate specimens. The specimens were dry-polished using emery paper up to #4000 and then buffed using an alumina suspension with grain sizes of 3 mm and 0.3 mm to obtain a mirror surface. After polishing, the specimens were ultrasonically cleaned with acetone and dried thoroughly.

### 2.2. Vickers Hardness Measurement

The Vickers hardness (*H_v_*) of each sample was measured using a Vickers hardness tester (HMV-2000, Shimadzu Co., Ltd., Kyoto, Japan). The test load was set to 4.93 N and the dwell time to 15 s. Twelve measurements were performed for each sample, and the average value was obtained from 10 points, excluding the maximum and minimum values of each sample, to obtain the Vickers hardness of each sample.

### 2.3. Sliding Wear Test

**Figure 1:**
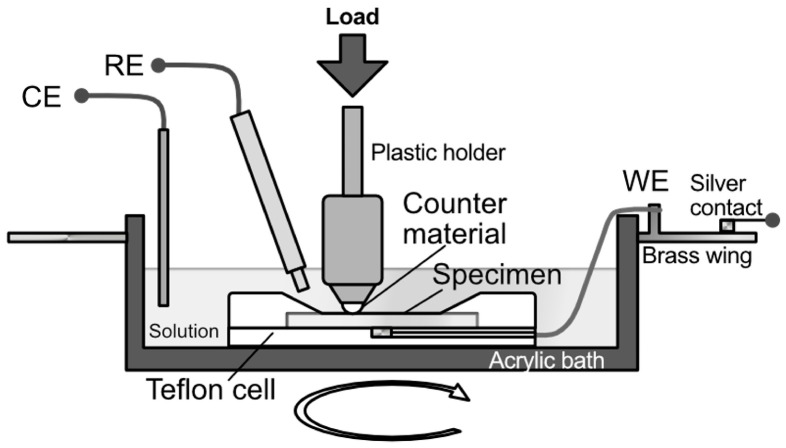
Schematic illustration of the experimental setup for the ball-on-disk in situ tribo-electrochemical test.

Sliding wear tests were performed using a ball-on-disk friction tester (FPR-2000S, RHESCA, Tokyo, Japan). A schematic of the tester is shown in Figure 1. Zirconia balls with a diameter of *f* 5 mm were used as counter materials. The friction and wear tests were conducted in ambient, in 100 mL of distilled water at 310 K or in 100 mL of HBSS at 310 K, a linear velocity of 20 mm/s, a turning radius of 4 mm, a turning speed of 47.7 rpm, a test time of 5.4 ks, and loading conditions ranging from 0.98 N to 5.88 N. Each of the wear tests was performed at least two times, and average values were plotted. For conditions where more than three times were taken, error bars of standard deviation were added to the plot.

After the wear test, the samples were immediately removed from the tribo-cell, ultrasonically cleaned in pure water and then acetone, and then dried in a vacuum desiccator. The volume-loss measurement function of a laser microscope (OLS3000, Olympus Co., Ltd., Tokyo, Japan) was used to measure the amount of wear. The cross-sectional area was determined using the step measurements after scanning the confocal image in the height direction to obtain a three-dimensional image.

The obtained wear volume was used to calculate the wear rate (*k*) [mm^2^/N] and specific wear rate (*k*′) [-] using the following equations:

(1a) $$k=\frac{V_{loss}}{PL}$$

(1b) $$k’=\frac{H_{v}V_{loss}}{PL}$$

where *V_loss_*, *P*, *L*, and *H_v_* are the total wear volume [mm^3^], dead load [N], wear distance [mm], and Vickers hardness [N], respectively. Here, Equation (1a) is based on the Holm equation and Equation (1b) is based on the Archard equation. Thus, *V_loss_* is defined by Equation (1c) by following Equation (1b).

(1c) $$V_{loss}=k’\frac{PL}{H_{v}}$$

The Holm and Archard equations are empirical laws that show that the amount of wear is proportional to the load and wear distance, and inversely proportional to the hardness [40,41,42].

### 2.4. Corrosion Volume-Loss Measurement

Corrosion volume-loss measurements were performed to quantify the material loss caused by immersion in distilled water or HBSS to determine the amount of volume loss caused by corrosion. The sample was then placed in a beaker filled with distilled water or HBSS at 310 K for 80 days. The beaker was continuously stirred in a shaker. From the weight change in the specimens obtained from the corrosion test, the volume loss in the wear scar due to corrosion was estimated using the following equation. After immersing the specimens in HBSS for 80 days and obtaining the weight change, the weight per unit area was calculated by dividing by the surface area and comparing it with the amount of wear, as shown in Equation (2).

(2) $$V_{loss}=A_{wear}\frac{\Delta m}{\rho A_{s}}$$

In this equation, *V_loss_*, *A_wear_*, Δ*m*, ρ, and *A_s_* are the volume loss [mm^3^], area of the wear track [mm^2^], weight change [g], density [g/mm^3^], and entire surface area of the sample [mm^2^], respectively.

### 2.5. In Situ Tribo-Electrochemical Measurement

The polarization curves of the Mg alloys were measured to investigate their corrosion characteristics during wear tests with different vertical loads in HBSS. Figure 1 shows a schematic illustration of the experimental setup for the in situ tribo-electrochemical tests used in the experiment. A Pt counter electrode and an Ag/AgCl reference electrode were placed in a tribo-cell, as shown in Figure 1. The reference electrode was placed as close as possible to the counter material. The sample was placed in a plastic cover cell except for the contact surface. The working electrode setup for the in situ tribo-electrochemical measurements is described below. A terminal in contact with the sample was placed on the back of the sample cover cell and a wire connected to a brass disk was placed at the edge of the rotating bath. The brass disk was in contact with a brush electrode connected to a potentio-galvanostat and a data logger, allowing potential measurements to be made while the bath was rotating. The potential manipulation range was set from −3.0 V to 3.0 V, and the potential sweep rate was 1 mV/s.

The pH change in the solution during the wear test was measured using a pH meter (TPX-999, Tokokagaku Co., Ltd., Kawasaki, Japan). The distance between the electrode and the sample was kept constant, and the measuring section was fixed as close to the sample as possible. The distance from the friction area was estimated as 15–20 mm.

### 2.6. Microstructure Observation of Wear Track

Scanning electron microscopy with X-ray energy dispersive spectroscopy (SEM-EDS; JSM-7001FA, JEOL Co., Ltd., Tokyo, Japan) was used to observe the microstructure of the worn surface. The chemical bonding state of each element was determined from the electron binding energy shifts measured using X-ray photoelectron spectroscopy using Al-Ka line (XPS, phi-5000, ULVAC phi Co., Ltd., Chigasaki, Japan). The binding energy of the measured spectrum was corrected by setting the peak position of the C-C peak of C 1s at 285 eV.

## 3. Results

### 3.1. Wear Volume Loss After Sliding Wear

**Figure 2:**
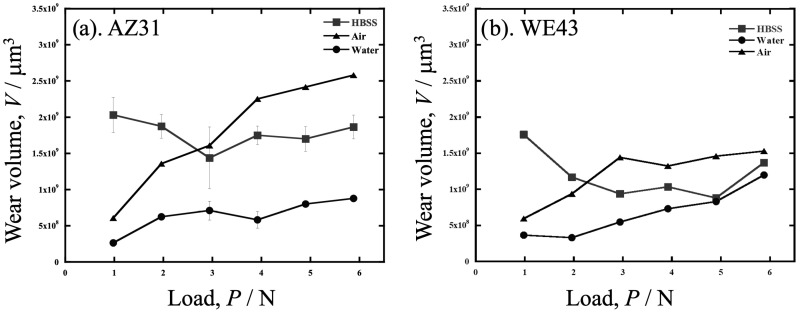
The relationships between the wear volume and dead load in (**a**) AZ31 and (**b**) WE43 under various atmospheres.

The relationships between the wear volume and dead load in AZ31 and WE43 under various atmospheres are shown in Figure 2. Furthermore, the Vickers hardness values of the polished surfaces of AZ31 and WE43 were *H_V_* = 62 and *H_V_* = 91, respectively. The wear volume of AZ31 in Figure 2 was the highest in water at 5.88 N. In the HBSS, the highest amount of wear was observed at 0.98 N. The amount of wear decreased with increasing load up to 2.94 N and then increased. In the low-load range (0.98 to 1.96 N), the wear rate was higher in HBSS, air, and water in that order, but in the high-load range (2.94 to 5.88 N), the wear rate was reversed between HBSS and air.

**Figure 3:**
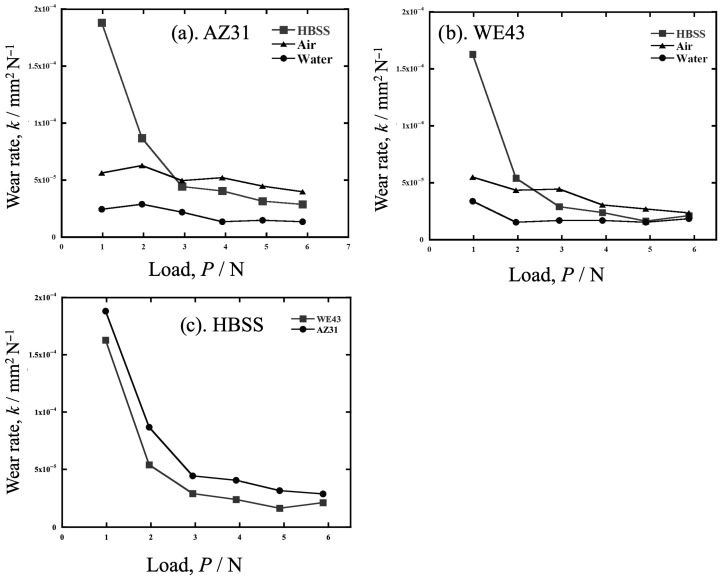
The wear rates *k* of (**a**) AZ31 and (**b**) WE43 tested in HBSS, in air and in water, and (**c**) the *k* of both alloys in HBSS.

Figure 3 shows the wear rates *k* of AZ31 and WE43 tested in HBSS, in air and in water, and the *k* of both alloys in HBSS. As shown in Figure 3a,b, the shape of the *k* versus the load (*P*) graph for AZ31 showed a similar trend to that for WE43 in all atmospheres. *k* was higher in air than in water, but in both atmospheres, *k* remained almost flat or decreased gradually with increasing load. In HBSS in Figure 3c, *k* was higher than in water at loads of 2.94 N or higher. As with the other atmospheres, *k* did not change significantly with increasing load. In particular, for WE43, *k* values were similar in all three atmospheres. At 1.96 N and 0.98 N, *k* increased with decreasing load.

**Figure 4:**
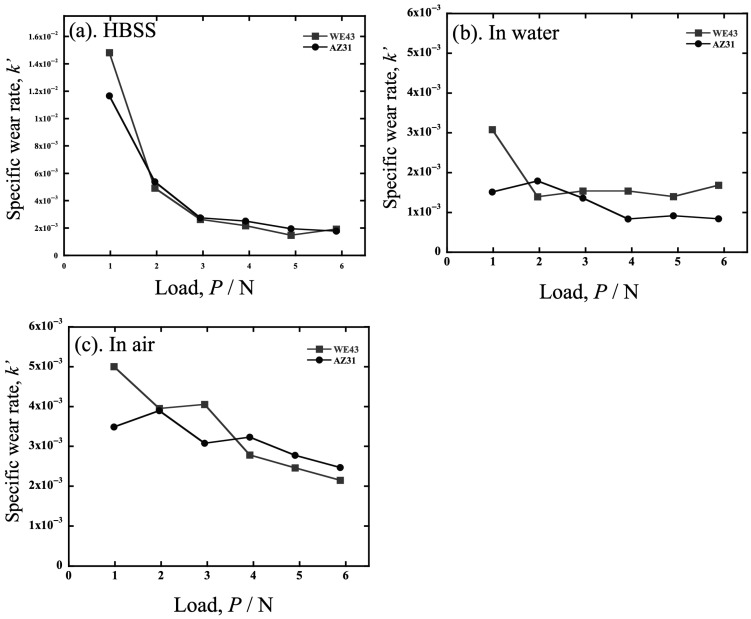
The specific wear rate, *k*′, of AZ31 and WE43 tested (**a**) in HBSS, (**b**) in air and (**c**) in water.

Another specific wear rate, *k*′, was obtained by multiplying the hardness (*H*_v_) and dividing it by *P* and wear distance (*L*). The results are shown in Figure 4. As shown in Figure 4a, the *k*′ values of AZ31 and WE43 in HBSS are almost identical, indicating that the wear difference between the alloys originated from the hardness. Similarly, when considering the results in water and air on the same *k*′ scale as HBSS, the *k*′ value of both alloys can be considered to be almost the same. In other words, the difference in wear volume between AZ31 and WE43 is thought to be mostly due to the difference in hardness.

**Figure 5:**
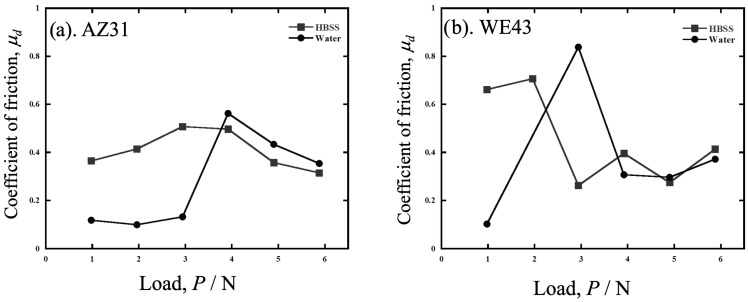
The dynamic frictional coefficient (*m_d_*) against the dead load of (**a**) AZ31 and (**b**) WE43 tested in HBSS and water.

Figure 5 shows a plot of the dynamic frictional coefficient (*m_d_*) against the load for wear in HBSS and water. In AZ31, the *m_d_* value was stable at low values of up to 2.94 N, but increased and decreased after 3.92 N. In WE43, *m_d_* was unstable below 2.94 N in water and HBSS, stabilizing at *m_d_*~0.3 as the load increased.

### 3.2. Worn Surface Observation of AZ31

**Figure 6:**
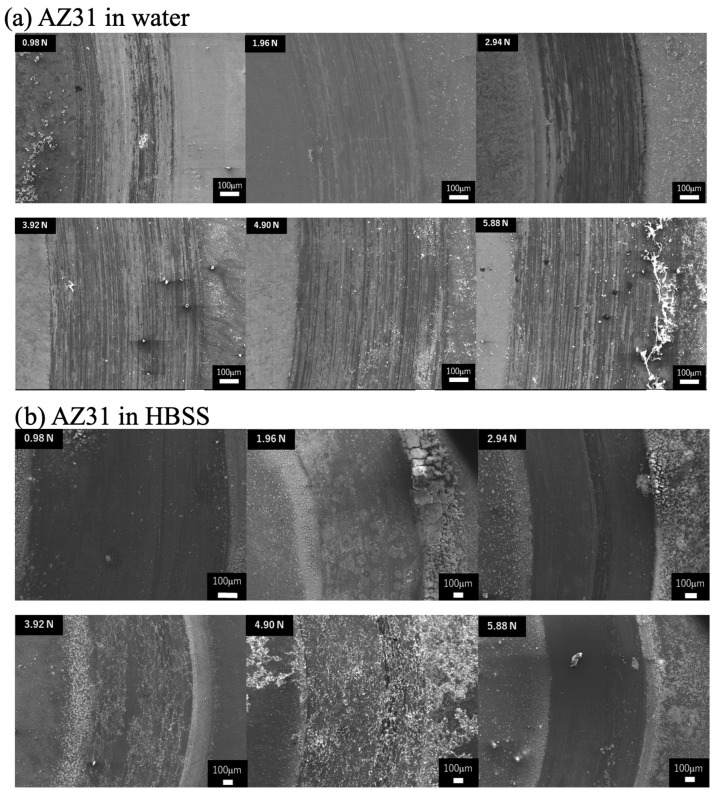
SEM images of worn surfaces of AZ31 alloy tested in (**a**) water and (**b**) HBSS with 0.98–5.88 N dead load.

Figure 6 shows the SE images of the wear scar of AZ31 tested at each load and in each lubricant. In the worn surface morphology tested in water (Figure 6a), clear-cut wear scars were observed at all loads. In addition, HBSS exhibited a smooth surface with fine and unclear wear scars, as shown in Figure 6b. It appears that the wear debris formation increased with increasing dead load in both lubricants. Furthermore, more debris was observed in the HBSS than in the water.

**Figure 7:**
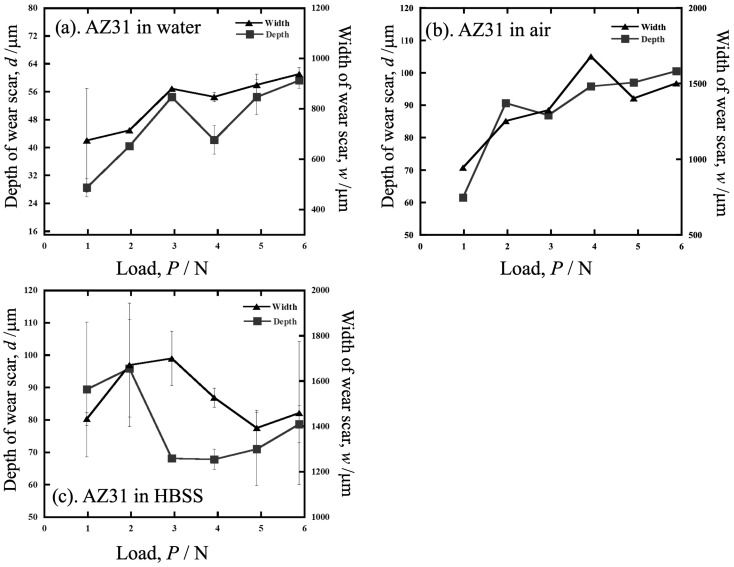
The average width and depth of AZ31 wear scars tested in water, air, and HBSS versus dead load.

Figure 7 plots the average width (*w* [mm]) and depth (*d* [mm]) of AZ31 wear scars tested in water, air, and HBSS versus load. In the results in water and air, both *w* and *d* tended to increase with increasing load, while in HBSS, both were higher at low loads up to 2 N. Depth was independent, and width decreased with increasing load above 3 N.

It is known that the dissolution reaction of Mg in water is:

(3) $$Mg+H_{2}O\to Mg{\left(OH\right)}_{2}+H_{2}$$

The dissolution rate of Mg in distilled water is lower than that in HBSS, where various salts, including carbonates, are present. The formation of Mg salts, such as Mg(OH)_2_, was also inferred to be low. In fact, in Figure 6b, mass residues of several tens to several hundreds of microns in size, which cannot be recognized as wear debris, were formed on the edge of the wear scar, and the surface condition of the wear scar suggests that the contribution of corrosion in addition to wear is significant in HBSS.

### 3.3. Corrosion Behavior

**Figure 8:**
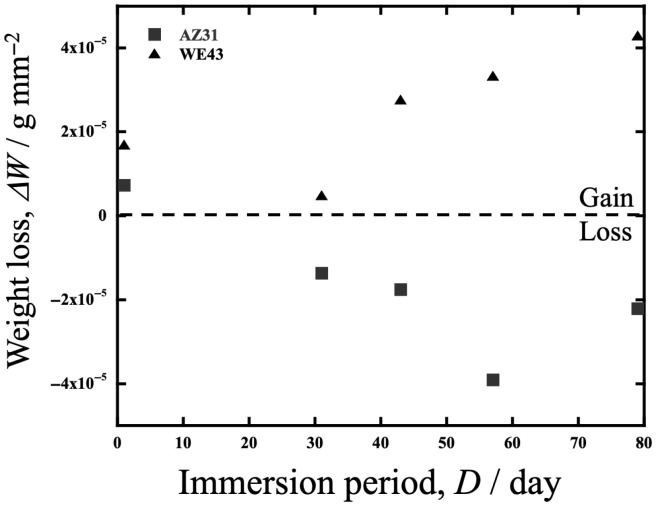
Weight change in AZ31 and WE43 against immersion time up to 6.91 Ms (80 days) in HBSS.

After the corrosion test, the specimen surfaces remained mirror-like except for AZ31 in HBSS, whose surface became slightly cloudy. Figure 8 shows a plot of weight change (*DW* [g/mm^2^]) against immersion time up to 6.91 Ms (80 days) in HBSS. The graph shows that weight gain occurred in WE43 and weight loss occurred in AZ31. This result in WE43 indicates that the formation of corrosion products, such as Mg(OH)_2_, was enhanced during immersion, which led to increased weight gain. Li et al. [21,43] reported that the corrosion rate of WE43 in SBF (simulated body fluid) is faster than that of AZ31. Although the details are discussed later, hydroxide formation from Mg dissolution is suggested to be more active in WE43 than in AZ31, resulting in a more pronounced weight gain. This dissolution-induced weight loss does not occur, as in AZ31.

In the presence of H^+^ and Cl^−^, OH^−^ reacts with H^+^ in a neutralization reaction, liberating Mg^2+^ ions from Mg(OH)_2_ to form soluble MgCl_2_.

(4) $$Mg{\left(OH\right)}_{2}(s)⇄Mg^{2+}\left(aq\right)+2{OH}^{-}\left(aq\right)$$

In the presence of Cl^−^ ions, the reaction in Equation (4) will be accelerated in the rightward direction. Therefore, it is expected that Mg(OH)_2_ is inherently difficult to form in HBSS. Furthermore, the corrosive reaction may be suppressed in AZ31 by the Al addition.

**Figure 9:**
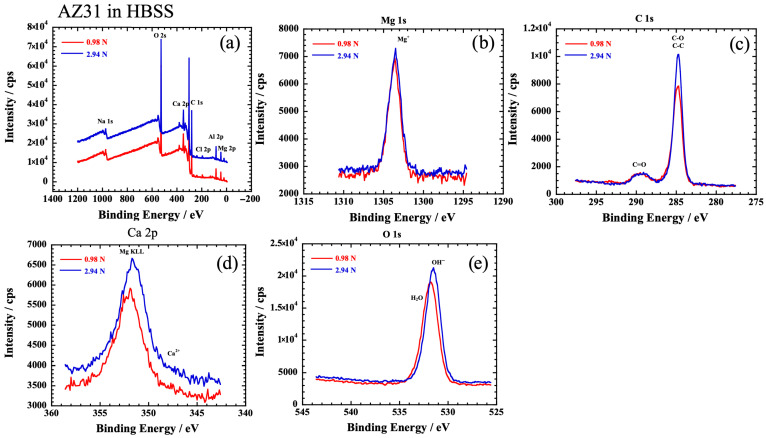
The XPS spectrum obtained within the wear scar area of AZ31 in HBSS. (**a**) Wide scan, (**b**) Mg 1s, (**c**) C 1s, (**d**) Ca 2p, (**e**) O 1s.

**Figure 10:**
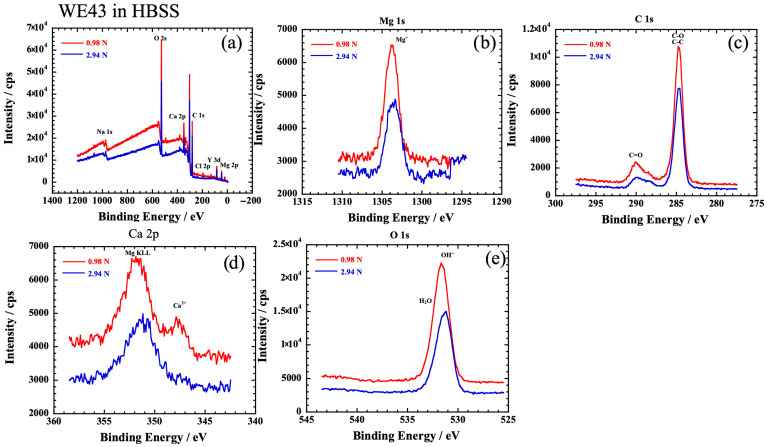
The XPS spectrum obtained within the wear scar area of WE43 in HBSS. (**a**) Wide scan, (**b**) Mg 1s, (**c**) C 1s, (**d**) Ca 2p, (**e**) O 1s.

XPS measurements were performed to compare the products of AZ31 and WE43 at vertical loads of 0.98 N and 2.94 N. Figure 9 and Figure 10 show the XPS spectrum obtained within the wear scar area of AZ31 and WE43 alloys, respectively. Peaks of Na, O, Ca, C, Cl, P, Al, and Mg were observed in the wide scan of AZ31, as shown in Figure 9a. The O 1s peak shifts indicate the presence of OH^−^ and H_2_O, and the Ca 2p peak in AZ31 was hardly detected at 347–351 eV. Similarly, Figure 10 shows the results of the XPS measurements in the wear scar of the WE43 alloy, with no difference except that phosphoric acid was hardly detected at 2.94 N. Peaks of Na, O, Ca, C, Cl, P, Y, and Mg were detected as shown in Figure 10. The presence of OH^−^ ions and Mg^2+^ ions in both alloys suggests that Mg(OH)_2_ is present on the surface, and the H_2_O peak is detected owing to moisture penetration in the HBSS. The C=O peaks in Figure 9c and Figure 10c suggest the presence of carbonate. There was no significant difference in the products among AZ31 at 0.98 N, at 2.94 N, and WE43 at 2.94 N. On the other hand, Ca compounds were relatively abundant in WE43 at 0.98 N. The H_2_O peaks were not detected, and the P 2p (130 eV) peak was also observed in the wide scan spectrum in WE43 at 0.98 N, suggesting the formation of calcium phosphate.

**Figure 11:**
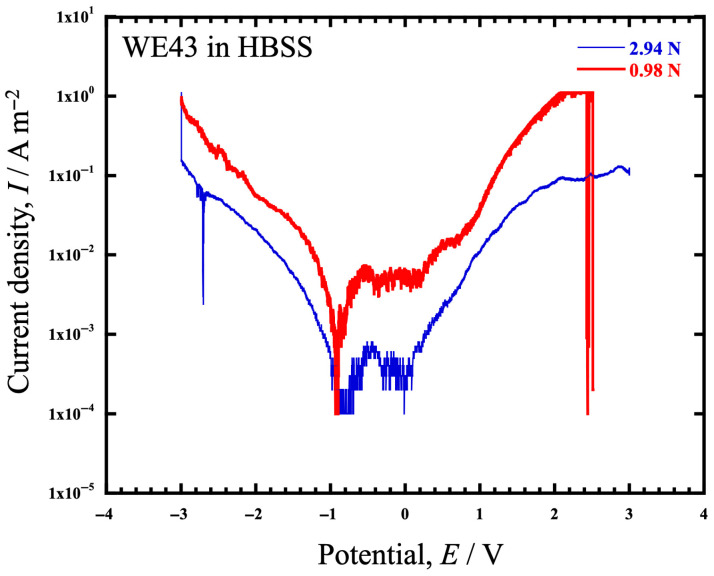
The anode polarization curves of WE43 in HBSS obtained by electrochemical measurements measured in the tribocorrosion cell at 0.98 N and 2.94 N.

Figure 11 shows the anode polarization curves obtained by electrochemical measurements measured in the tribocorrosion cell with a dead load, and Table 3 shows the summary of the electrochemical parameters, corrosion potential (*E*_corr_), corrosion current density (*I*_corr_), passive current density (*I*_p_), and pitting potential (*E*_pit_). In these figures and tables, *E*_corr_ at 0.98 N was shifted approximately 0.07 V to the lesser side compared to that at 2.94 N, and *I*_corr_ and *I*_p_ at 0.98 N were an order of magnitude higher than that at 2.94 N, suggesting that anodic dissolution is accelerated at 0.98 N and corrosion can occur more easily at 0.98 N. This result suggests that the magnitude of the dead load affects the electrochemical reaction on the sample surface in some way.

## 4. Discussion

### 4.1. Wear in Water

When the amount of wear volume in water, air, and HBSS is compared as shown in Figure 2, the wear volume of both alloys in water is the smallest compared to HBSS and air for the entire load. In addition, both wear volumes were approximately the same. Conversely, regarding the lubricant effects on the alloys, the difference between the alloys in HBSS and in air is more significant than in water; that is, the wear volume of AZ31 is more significant than that of WE43 in HBSS and air, especially at higher load ranges above approximately 3 N. The difference in wear volume in air is thought to result from WE43 being a harder alloy than AZ31, as shown in the results for *k*′ in Figure 4.

Table 4 shows the quantification results with SEM-EDS of the WE43 wear samples in the water outside the wear scar. The high oxygen content indicates that magnesium hydroxide, a corrosion product of Mg, was formed on the sample surface owing to water immersion. It is known that in the presence of a small amount of water, the corrosion resistance of the specimen surface increases because of the formation of a Mg(OH)_2_ gel film on the surface [6,44,45]. Thus, this gel film may have played a protective role against the wear and corrosion of WE43 in water. This protective function of the gel film against wear also affects the wear of AZ31. Therefore, it is considered that there was no significant difference in wear volume between the alloys, which had different hardness values, because the protective gel film formed in water during the wear test acted as a lubricant.

### 4.2. Wear in HBSS

#### 4.2.1. Relationships Between Applied Load and Tribocorrosion

The XPS results showed that Mg(OH)_2_ was observed in the wear traces in HBSS after the wear test, indicating that the protective film also formed in HBSS, similar to water. However, although not as pronounced as in air, the wear volume in WE43, which has a higher hardness, is lower than that of AZ31. These results suggest that the protective effect of the Mg(OH)_2_ gel film in HBSS was weaker than that in water. Cl^−^ ions have been suggested as the reason for the weakening of this effect [43,46]. Previous studies have reported that the Mg(OH)_2_ film is dissolved by Cl^−^ ions to form a MgCl_2_ salt, decreasing the corrosion resistance [43,46]. Thus, it is reasonable that Cl^−^ in HBSS prohibits the formation of a protective film.

**Figure 12:**
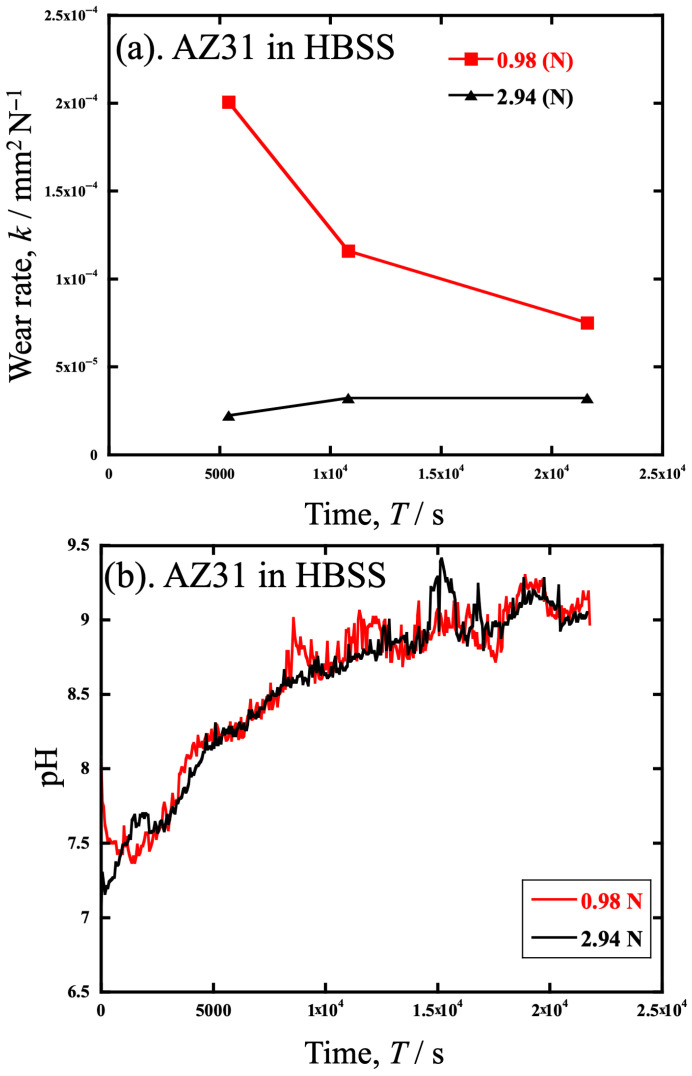
The specific wear *k* of AZ31 in HBSS and pH change in the solution during the test at loads of 0.98 N and 2.94 N. (**a**) *k* and (**b**) pH were measured simultaneously in the cell.

Figure 12 shows a graph of the specific wear *k* of AZ31 in HBSS and pH change in the solution during the test at 0.98 N and 2.94 N. Both measurements were performed simultaneously in the cell. In Figure 12a, *k* at 0.98 N decreases with test time, whereas *k* at 2.94 N almost plateaus. Interestingly, *k* at 0.98 N is higher than at 2.94 N, which means that load-independent wear occurred. In other words, Holm’s law in Equation (1c) means *k* should plateau when the volume loss is due to wear. This result suggests that the volume loss is mainly due to wear at 2.94 N, but not 0.98 N. Other effects, such as corrosion wear, should be significant during the wear test at 0.98 N.

Furthermore, Figure 12b shows the time dependence of the pH in HBSS during the wear test. It can be seen that the pH increases similarly with time, either at 0.98 N or 2.94 N. Only at 0.98 N does the specific wear decrease with increasing pH. The fact that *k* at 0.98 N decreases with time while not changing at 2.94 N probably indicates that corrosion has a more significant effect at 0.98 N but not at 2.94 N. This is consistent with the anodic polarization curve obtained in Figure 11, which shows that 0.98 N is less corrosion resistant.

The results of Figure 11 and Table 3 suggest that WE43 exhibits similar corrosion wear characteristics to AZ31, depending on the load. As shown in Table 3, increasing the load slightly shifted *E*_corr_ to the precious side, and *I*_corr_ and *I*_p_ decreased by an order of magnitude. If the contribution of corrosion is stronger at 0.98 N than at 2.94 N, then there should be more Mg(OH)_2_ generation; however, it should be taken into account that the *I*_corr_ increases because the mechanical sliding constantly removes the Mg(OH)_2_ protective layer, exposing “fresh” reactive Mg to the HBSS. While the polarization curves in Figure 11 show particularly high noise in the anode region, it is likely due to the presence of a stress distribution by the load, and the resulting non-uniformity of the corrosion product distribution such as Mg(OH)_2_. They provide a qualitative comparison of the electrochemical activity under different loading conditions. This trend suggests that while potentiodynamic polarization captures the overall trend, the localized kinetics at the sliding interface are highly complex.

#### 4.2.2. Lubricating Components That Most Affect Corrosive Wear

**Figure 13:**
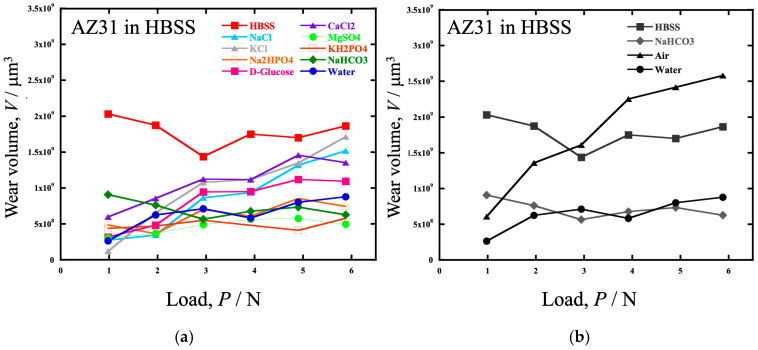
The load dependence of the volume loss in the AZ31 alloy tested in (**a**) solutions containing each component of HBSS (NaCl, KCl, Na_2_HPO_4_, D-glucose, CaCl_2_, MgSO_4_, KH_2_PO_4_, NaHCO_3_), water, HBSS and (**b**) in HBSS, NaHCO_3_ aq., air and water.

To specify the HBSS component most relevant to the corrosion wear of the Mg alloys, wear tests were conducted in a solution of each component of HBSS. Figure 13a shows the load dependence of the volume loss in the AZ31 alloy. The wear volumes in each component solution were lower than those in HBSS. The volume loss in NaCl, KCl, D-glucose, or CaCl_2_ solutions was more significant than that in water, and in all components except NaHCO_3_, volume loss tended to increase with increasing load. It can be considered that Cl^−^ ions in the NaCl, KCl, and CaCl_2_ solutions decreased Mg(OH)_2_ film formation, resulting in increased wear volume. It has also been reported that an increase in the D-glucose concentration in saline solution increases the corrosion rate of pure Mg [47]. In addition, the effect of pH buffers also affects corrosion wear. Carbonate and phosphate ions have a buffering effect and the pH of the solution affects the corrosion rate.

The only solution that showed an increase in the wear volume below 3 N, similar to HBSS, was NaHCO_3_. Figure 13b shows a plot comparing the wear volume in NaHCO_3_ with that in HBSS, water, and air. However, the amount of wear in the NaHCO_3_ solution was almost consistent with that in water. This is thought to be due to the improvement in corrosion resistance and wear resistance by film formation, as it has been reported that immersion of Mg and Mg alloys in NaHCO_3_ solution improves corrosion resistance and is effective for surface modification [48,49].

**Figure 14:**
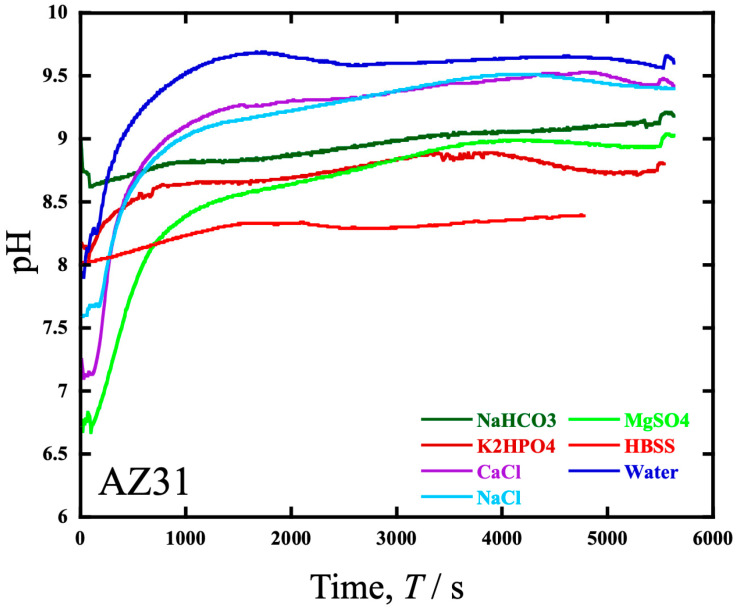
The time variation in the pH during the wear test of AZ31 in various solutions containing each component of HBSS (NaHCO_3_, KH_2_PO_4_, CaCl_2_, NaCl, MgSO_4_), HBSS, and water.

Figure 14 shows the time variation in the pH during the wear test in various solutions. The pH electrode was placed at a distance from the sample, that is, along the sidewall of the rotating cell. The distance from the friction area was approximately 40 mm. The results in Figure 14 show that the HBSS and NaHCO_3_ solutions, which showed a similar wear rate load dependence, had a pH difference between 8 and 8.75. However, they show a similar pH curve with a constant increase from the initial value, although pH drastically increased with time in other solutions up to 1.5 ks.

### 4.3. Mechanism of Tribocorrosion

This section discusses the causes of the increase in specific wear in the low-load range below 3 N. Based on the results obtained in this study, the plastic deformation of the contact surface should be considered. The maximum Hertzian contact pressure *s_Hmax_* can be estimated using the literature value, and the obtained *s_Hmax_* of AZ31 was approximately 557 MPa for a load of 2.94 N and 386 MPa for a load of 0.98 N. According to the literature [50], the 0.2% proof stress of AZ31 extrusions under compression is approximately 100 MPa. In addition, the Young’s modulus of AZ31 is approximately 40–45 MPa [51,52,53], whereas that of the counter face, ZrO_2_ ball, is approximately 200–220 MPa [54,55]; AZ31 is exclusively deformed under load. Thus, the contact area of the sample was plastically deformed even at 0.98 N during the test.

It should be noted that the wear volumes measured in this study represent the net topographical change in the surface. While corrosion products, such as Mg(OH)_2_, likely adhered to the wear scar during measurement, the volume loss at 0.98 N remains significantly higher than at 2.94 N, despite the potential filling effect of these products, further underscores the severity of the material dissolution. If all corrosion products were completely removed, the calculated material loss at low loads would likely be even more pronounced since corrosion was enhanced at low load, reinforcing the dominance of the tribocorrosion mechanism in this regime.

The reason for the increase in *k* at 2.94 K or less in HBSS is thought to be the influence of the surface condition; however, this alone cannot explain the effect of the dead load. According to previous calculations of the contact pressure, when a dead load is applied, the surface of the material undergoes elastic and plastic deformation according to the dead load. This causes the surface to sink, and a gap forms between the surface and the counter ball. The gap size should differ depending on the load.

**Figure 15:**
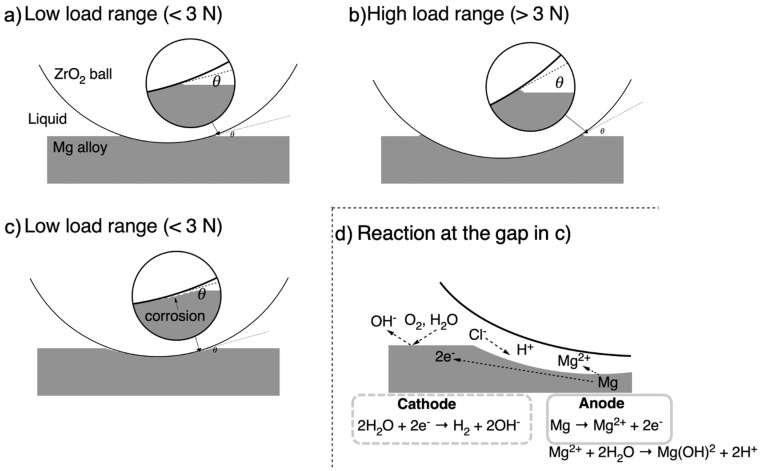
The schematic of the concentration cell model based on the results obtained in this study.

A schematic of the concentration cell model based on the results obtained in this study is shown in Figure 15. A gap should be generated between the ZrO_2_ ball and the contact surface of AZ31, which has sunk owing to the difference in the shape and deformation capacity of the specimen and the counter material. When the diameter of the ZrO_2_ ball i constant, the size of this gap depended on the sinkage of the sample, that is, with the load, as shown in Figure 15a,b. The gap height near the contact point was small when the load was small, and the ball sank at a small depth, as shown in Figure 15a. In addition, when the solution and the ball are sufficiently wet and the solution viscosity is sufficiently small, the solution is more likely to penetrate the gap due to capillary action. For an aqueous solution in which various ions, including Cl^−^, are present, the concentration distribution of Cl^−^ ions between the gap and the rest of the solution causes the H^+^ in the gap to become concentrated, subsequently forming a concentrated battery. Because the anodic gap area is narrower than the other cathodic areas, the corrosion current concentrates in the gap, accelerating corrosion in the gap area. This means that crevice corrosion occurred at the gap, as shown in Figure 15d.

This mechanism is further supported by the SEM observations in Figure 6b, where prominent mass residues were identified specifically at the edges of the wear scars in HBSS. These residues likely represent the localized accumulation of corrosion products in the stagnant zone of the crevice. The intensity of this effect at low loads can be attributed to the specific gap geometry; at lower loads, the elastic sinking of the substrate is shallow enough to maintain a narrow, stable crevice that promotes electrolyte entrapment and oxygen depletion. Conversely, as the load increases, the deeper penetration of the ZrO_2_ ball and the increased mechanical sweeping action likely disrupt the stability of this concentration cell, effectively venting the crevice and shifting the primary degradation mode from chemically dominated tribocorrosion toward mechanical wear.

When localized corrosion occurs at the edge of the wear scar, where a gap forms, only the edge of the wear scar leaches out as the wear scar spreads. The contact area between the specimen and counter material narrows at the center, resulting in a more substantial pressure. The increased pressure due to the smaller contact area caused deeper wear. As a result, it was considered that the wear scar width and depth increased in the low-load range, and the wear loss volume increased, increasing the amount of wear.

The plot of the pH changes over time during the wear test in each aqueous solution in Figure 14 shows that, except for the wear in the HBSS and NaHCO_3_ solutions, rapid pH increases occur in the initial stage of immersion, whereas in the HBSS and NaHCO_3_ solutions, the overall pH value remains constant at 8–9. Therefore, it is considered that a difference in pH may occur between the solution and the vicinity of the wear scar, causing corrosion similar to crevice corrosion that occurs continuously and contributes to an increase in the amount of wear.

However, because the cell is rotating, the friction force received on a part with the wear surface is intermittent when considering the effect of the solution velocity and the frictional force received on a single point. Because crevice corrosion is a time-dependent reaction, it remains to be examined whether the crevice corrosion mechanism can be directly applied to sliding wear. However, in terms of this phenomenon, the results obtained in this study are well-adapted to this mechanism.

## 5. Summary and Conclusions

Sliding wear tests were conducted on AZ31 and WE43, considered for biodegradable medical implants, in distilled water and aqueous solutions of HBSS at 310 K and ambient atmosphere. The load dependence of the wear parameters was obtained to investigate the relationship between the amount of wear, load, atmosphere, surface morphology, surface chemical analysis, and electrochemical measurements, focusing on the load dependency of the wear parameters. In addition, to identify the components that cause tribocorrosion, we measured the load dependence of specific wear in each element of the HBSS. The following conclusions were drawn:

(1) Comparing the load dependence of wear in air, deionized water, and HBSS, the wear of both the AZ31 and WE43 alloys was greater at loads above 2 to 3 N in the order of air > HBSS > water. Below 2 to 3 N, the wear of HBSS increased and was the largest. In the comparison between the alloys, the amount of wear in water was similar at each load; however, the increase in the amount of wear with increasing load was more pronounced for AZ31, that is, AZ31 showed a stronger load dependence.
(2) However, when converted into a specific wear rate, the specific wear of AZ31 and WE43 was not significantly different at any load, and the effect of the atmosphere or liquid was also small, except for HBSS.
(3) For AZ31 and WE43, the specific wear rate in water was smaller than that in air and HBSS. This was due to the improved corrosion and wear resistance resulting from the formation of a protective Mg(OH)_2_ gel. The Cl^−^ ions in HBSS prohibit gel formation.
(4) The wear rate in HBSS increased drastically with a load decrease of less than 3 N. This result indicates that the wear in HBSS is affected by factors other than the load, running distance, and hardness.
(5) Electrochemical measurements of WE43 in HBSS showed that the polarization behavior differed between a load of 0.98 N and 2.94 N, with a tendency for corrosion to occur more likely at 0.98 N.
(6) Similar to the wear in HBSS, the wear in NaHCO_3_ aq. exhibited a tendency to increase in the low-load range. The amount of wear also increased under Cl^−^ and D-glucose conditions, which significantly influenced the increase in volume loss.

The results obtained in this study suggest that the local dissolution of wear scar edges due to crevice corrosion and the reduction in contact area are related to the increased wear in HBSS at a low-load range.

## Figures and Tables

**Table 1:** The chemical compositions of AZ31 used in this study.

	Al	Zn	Mn	Fe	Si	Cu	Ni	Mg
mass%	2.76	0.84	0.2708	0.0024	0.0116	0.0014	0.0007	bal.

**Table 2:** The chemical compositions of WE43 used in this study.

	Zn + Ag	Y	Cu	Mn	Fe	Nd	Zr	RE	Mg
mass%	0.03	4.00	0.002	0.01	0.001	2.3	0.48	1.1	bal.

**Table 3:** The list of the electrochemical parameters (*E*_corr_, *I*_corr_, *I*_p_, *E*_pit_) obtained from the anode polarization curves of WE43 in HBSS at 0.98 N and 2.94 N in Figure 11.

Test Load (N)	*E*_corr_ (V)	*I*_corr_ (A/m^2^)	*I*_p_ (A/m^2^)	*E*_pit_ (V)
0.98	−0.929	5.58 × 10^−4^	5.21 × 10^−3^	0.218
2.94	−0.853	5.90 × 10^−5^	3.44 × 10^−4^	0.083

**Table 4:** The chemical compositions measured by SEM-EDS of the outside of the wear scar of the WE43 sample after the wear test in water.

	O	Na	Mg	P	Ca	Y	Nd	C
mol%	25.74	nd	57.37	1.59	nd	4.03	2.1	9.17

## Data Availability

The original contributions presented in this study are included in the article. Further inquiries can be directed to the corresponding author.
